# Evaluation of anticancer, antioxidant and antibacterial properties of methanol extract of three* Acantholimon* Boiss. species 

**Published:** 2020

**Authors:** Sara Soltanian, Mahboubeh Sheikhbahaei, Mansour Mirtadzadini, Behjat Kalantari Khandani

**Affiliations:** 1 *Department of Biology, Faculty of Science, Shahid Bahonar University of Kerman, Kerman, Iran*; 2 *Department of Internal Medicine, School of Medicine, Kerman University of Medical Sciences, Kerman, Iran*

**Keywords:** Acantholimon austro-iranicum, Acantholimon serotinum, Acantholimon chlorostegium, Anticancer, Antioxidant, Antibacterial

## Abstract

**Objective:**

*Acantholimon* is a genus of perennial plant within the Plumbaginaceae family. Here, we aimed to investigate anticancer, antioxidant, and antibacterial potential of methanol extract of three Iranian endemic species of *Acantholimon* including *A. austro-iranicum, A. serotinum* and *A. chlorostegium. *

**Materials and Methods:**

MTT assay was used to evaluate the *in vitro* cytotoxicity and apoptosis induction was examined by annexin V-PE apoptosis detection kit. Antioxidant activity was reported based on the DPPH-scavenging and DCF-DA assay. Antibacterial activity was measured by disc diffusion and micro-well dilution assay.

**Results:**

MTT assay showed less cytotoxicity of methanol extracts against the HUVEC normal cell line (IC_50_ values: 817-900 µg/ml) compared to cancer cell lines MCF-7, HT29, SH-SY5Y, NCCIT and A549 (IC_50_ values: 213 to 600 µg/ml) that show the specificity of extracts toward cancer cells. Plant extract showed apoptosis induction and cell cycle arrest at the G0/G1 phases documented by annexin V staining and flow cytometry. According to antioxidant tests, extracts exhibited significant DPPH scavenging potential (IC_50_ values: 30-37 µg/ml) and could protect against H_2_O_2_-induced oxidative stress. Antibacterial activities showed a stronger inhibitory effect on *Escherichia coli* and *Pseudomonas aeruginosa *as Gram- negative bacteria (diameter of inhibition zone: 11-13 mm and minimal inhibition concentration (MIC): 3.175 to 12.5 mg/ml) compared to Gram-positive bacteria including *Enterococcus faecalis* and *Staphylococcus aureus *(diameter of inhibition zone: 3-7 mm and MIC: 25 to 50 mg/ml).

**Conclusion:**

Our results suggested moderate cytotoxic and antibacterial potential and noteworthy antioxidant activity for the examined *Acantholimon* species.

## Introduction

Recently, medicinal plants have been recognized as a source of biologically active compounds with therapeutic potential and many anti-cancers drugs, antibiotic prototypes and antioxidants have natural sources (Atanasov et al., 2015 ▶; Newman and Cragg, 2016 ▶; Pinto and Silva, 2017 ▶; Thomford et al., 2018 ▶). Plant-derived anticancer drugs can reduce the side effect of chemotherapy drugs on normal cells and improve efficiency of chemical therapeutic agents (Greenwell and Rahman, 2015 ▶; Sameer et al., 2016 ▶; Pinto and Silva, 2017 ▶). Many plant-derived secondary metabolites also have antimicrobial properties (Cowan, 1999 ▶; Dahanukar et al., 2000 ▶; Sher, 2009 ▶). Moreover, many types of natural antioxidants are available in medicinal plants that by scavenging or stabilizing free radicals, can reduce oxidative stress and its adverse effects on human health (Sreejayan and Rao, 1996 ▶; Gerber et al., 2002 ▶; Matteo and Esposito, 2003 ▶). Consequently, medicinal plants can be considered a rich source for herbal drugs for prevention and treatment of many diseases.

Plumbaginaceae is a family of flowering plants that is composed of 30 genera and about 725 species (Christenhusz and Byng, 2016 ▶). In this family, the genus *Acantholimon Boiss* consists of more than 200 species which are distributed in many different areas of the Irano-Turanian region (Kubitzki, 1993 ▶; Hernández-Ledesma et al., 2015 ▶; Lashgari et al., 2016 ▶; Nasiri et al., 2016 ▶; Gazor et al., 2017 ▶).

Although, pharmacological activities of certain types of the Plumbaginaceae were investigated (Kuo et al., 2002 ▶; Chaung et al., 2003 ▶; Eren, 2016 ▶), considerable research was not done on biological activities and medical use of *Acantholimon* as a large genus. The present work is the first study dealing with cytotoxicity, antioxidant and antimicrobial potential of methanol extract of *A. austro-iranicum, A. serotinum* and *A. chlorostegium* as endemic plants distributed in the south-east of Iran. 

## Materials and Methods

**Plant material**

Aerial parts of the following species were gathered, deposited in MIR herbarium and identified according to the standard keys. 

*A. austro-iranicum. *Iran. South-East, Kerman Province, Baft to Khabr, Mirtadzadini 1969 (MIR)

*A. serotinum, *Iran. South-East, Kerman Province, Baft to Khabr, Mirtadzadini 1968 (MIR)

*A. chlorostegium, *Iran. South-East, Kerman Province, Baft to Khabr, Mirtadzadini 2043 (MIR) 

**Preparation of the methanol extracts**

The aerial parts (150 g) of *Acantholimon* were dried in shade and extracted using 80 % methanol (500 ml) by continuous shaking at 200 rpm for 72 hr. 

After vacuum filtration, extracts were concentrated to obtain semisolid extracts at a maximum temperature of 40ºC using a rotary evaporator (EYELA SB-1100, JAPAN). The methanol extracts were concentrated to dryness in the oven at 40ºC. The dried extracts were dissolved in DMSO to make a stock of 250 mg/ml and further diluted to a final concentration of 3 mg/ml using complete culture medium. 

**Cell culture**

MCF-7 (human breast cancer), HT29 (human colon cancer), SH-SY5Y (human neuroblastoma), NCCIT (human embryonic carcinoma), A549 (human non-small-cell lung cancer) and HUVEC (human umbilical vein endothelial) cell lines were purchased from Pasteur Institute cell bank (Tehran, Iran). MCF-7, HT29, SH-SY5Y and HUVEC were maintained in Dulbecco’s Modified Eagle’s Medium (DMEM; Gibco), NCCIT and A549 were cultured in RPMI 1640 (Gibco). All media were supplemented with 10% fetal bovine serum, 100 U/ml penicillin, and 100 µg/ml streptomycin. These cells were kept in a humidified atmosphere containing 5% CO_2_ at 37ºC. 

**Cell viability assay**

Different cancer cell lines including MCF-7, HT29, SH-SY5Y, NCCIT and A549 were treated with different concentration of the methanol extract of *A. austro-iranicum, A. serotinum* and *A. chlorostegium* for 48 h and cytotoxic activity was evaluated using MTT assay. HUVEC human umbilical vein endothelial cell line was selected to examine the toxic effect of the extracts on normal cells. Briefly, 5×10^3 ^cells were seeded in 96-well tissue culture plates for overnight incubation and treated with increasing concentrations of the extracts (10 to 1250 μg/ml) for 48 hr. The cells were then incubated with medium containing MTT (3-(4, 5-dimethyl-2-thiazolyl)-2, 5-diphenyl-2H-tetrazolium bromide) at a final concentration of 0.5 mg/ml for 3 hr. After this period, media was removed and 100 µl DMSO was added to each well to dissolve formazan crystals and finally, enzyme-linked immunosorbent assay (ELISA) reader (BioTek-ELx800, USA) at 490 nm was used to measure optical densities (ODs). Mean absorbance for each concentration was divided to the mean absorbance of its controls (control samples were incubated with an equivalent amount of DMSO as the solvent of plant extracts) to calculate percentage of cell viability. The IC_50_ values of each extract were calculated using Prism 6.0 (Graph Pad Software, Inc., San Diego, California, USA).

**Colony formation assay**

The inhibitory effect of plant extract on cellular proliferation was also determined using a colony-forming assay. NCCIT cells were plated in 6well dishes at 1x10^3^ cells per well and treated with various concentration (25, 50, 100, 200 µg/ml) of *A. serotinum* methanol extract for 14 days. The colonies were fixed with methanol and stained with crystal violet. Stained colonies containing>50 cells were counted and plating efficiency was calculated according to the following formula: colony number/total cell number) × 100%.

**Apoptosis assay by flow cytometry**

Apoptosis assay was performed on NCCIT cells treated with *A. serotinum* methanol extract. Briefly, cells at a density of 3×10^5 ^were grown in 60-mm petri dishes and allowed to attach for 24 hr and then they were treated with 700 μg/ml of the plant extract. After 24 hr incubation, cells were washed with phosphate buffered saline (PBS) and centrifuged at 300 g for 5 min. Afterward, the cells were suspended in 100 μl annexin V binding buffer and 5 µl of PE Annexin V and 5 µl of 7-AAD and incubated in the dark at room temperature for 15 min. Annexin/7-AAD was evaluated by Becton Dickinson FACScan instrument using fl2 and fl3 filters for detection of annexin-PE and 7-AAD. 

**Cell cycle analysis**

NCCIT cells (5x10^6^) were seeded into 60 mm dishes and subjected to 300 µg/ml of *A. serotinum *extract for 72 hr. Adherent cells were detached by trypsinization and washed twice with PBS. Cells were fixed using 70% ice-cold ethanol for 30 min and incubated with 20 µg/ml propidium iodide and 10 μg/ml RNase A for 1 hr at 37°C in the dark. The stained cells were subsequently analyzed using BD FACSCalibur flow cytometer at a wavelength of 488 nm, equipped with Cell Quest 3.3 software.

**DPPH free radical scavenging assay**

To determine the antioxidant activity of methanol extract of three *Acantholimon *species, 1, 1-diphenyl-2-picrylhydrazyl (DPPH) radical scavenging assay was used. The antioxidant agents can convert DPPH with purple color into the yellow molecule, 1-1diphenyl-2-picryl hydrazine by donating electron or hydrogen (Thambiraj et al., 2012 ▶). In detail, the extract (50 μl) at concentrations ranging from 1.5 to 2000 µg/ml was added to 150 μl of 0.04 mg/ml DPPH (Sigma–Aldrich; St-Louis, USA) solution in methanol and incubated in the dark for 30 min. DPPH in methanol without the extracts served as a control. The blank was prepared in the same manner except that methanol was used instead DPPH solution to eliminate interference in each sample. The reduction of DPPH absorbance was measured at 515 nm using a plate reader (BioTek-ELx800, USA). Butylated hydroxytoluene (BHT) was used as a positive control. All determinations were performed in triplicate. 

The percentage inhibition (I%) was calculated in the following way: I%= [Absorbance of control-(Absorbance of sample-Absorbance of blank)]/Absorbance of control×100. The concentration of the plant extract that scavenge 50% of the total DPPH radical (IC_50_ value) was calculated from the graph plotting inhibition percentage against the extract concentrations. 

**Intracellular reactive oxygen species scavenging activity**

Intracellular reactive oxygen species (ROS) levels of living cells were determined using 2′, 7′-dichlorodihydrofluorescein diacetate (DCF-DA; Sigma-Aldrich, USA). DCFH-DA can be deacetylated by cellular esterases to the non-fluorescent DCFH. DCFH is oxidized to fluorescent DCF (2′, 7′-dichlorofluorescein) in the presence of ROS, which can be readily detected by a spectrofluorometer. Briefly, 25×10^3^ NCCIT cells were cultured in 96-well microplates for 24 hr. Then, the medium was removed and cells were exposed to PBS containing 20 μM of DCFH-DA (Sigma-Aldrich, USA) and kept in a humidified atmosphere (with 5% CO_2_ at 37°C) for 45 min. Next, cells were treated with H_2_O_2_ (200 μM, Sigma-Aldrich, Germany) in the absence/presence of each plant extract (50, 100, 200, and 400 µg/ml). After 3 hr, the fluorescence intensity was quantified using a fluorescence plate reader at an excitation of 485 nm and an emission of 538 nm (FLX 800; BioTek).

**Antimicrobial activity**

**Bacterial strains**

The antibacterial potency of *A. austro-iranicum, A. serotinum* and *A. chlorostegium* methanol extracts was individually examined against 4 bacteria strains that were obtained from Iranian Biological Resource Center. *Enterococcus faecalis* (ATCC 29212) and *Staphylococcus aureus* (ATCC 25838) as Gram-positive strains and *Pseudomonas aeruginosa *(ATCC 27853) and *Escherichia coli* (ATCC 11333) as Gram-negative strains, were tested in this study. 

**Disc diffusion method**

Antibacterial potential of methanol extracts was determined by agar disc diffusion method (Lehmann, 1999 ▶). The dried plant extracts were dissolved in DMSO to a final concentration of 200 mg/ml. Bacterial strains were sub cultured from original culture stored at -80ºC and grown on Mueller-Hinton Broth at 37°C for 24 hr. To obtain a lawn culture, 100 µl of the bacterial suspension containing 10^8^ CFU/ml of bacteria was spread on Mueller Hinton agar. The 6 mm disc impregnated with extract solution (200 mg/ml), DMSO (as negative control) and ciproﬂoxacin (25 mg/ml, as positive control) were placed on the lawn cultures. After 24 hr incubation at 37ºC, antibacterial activity was measured as the diameter of the inhibition zone produced by the extract around the disc.

**Determination of minimal inhibition concentration** (**MIC)**

MIC values were calculated for bacterial strains using micro-well dilution assay method (Baron, 1999 ▶). Three replicates of the serial two-fold dilutions of each extract (0.003 to 200 mg/ml) were prepared in Mueller Hinton Broth medium in 96-well plates. Next, 10 µl of bacterial suspension (10^8^ CFU/ml) was added into each well. The plates were placed in an incubator at 37°C for 24 hr. The wells containing 200 µl of the culture media and 10 µl of bacterial suspension without the test materials, were used as the negative control. The microorganism growth was shown by turbidity and the lowest concentration of the plant extracts required for inhibiting the growth of microorganisms (MIC value) was obtained. All tests were repeated twice. 

**Minimum bactericidal concentration (MBC)**

MBC value is defined as the lowest concentration of extract, at which no growth was observed. MBC was measured after MIC determination. It is determined by sub culturing broth dilutions that inhibit growth of a bacterial organism (i.e., those at or above the MIC). The broth dilutions are streaked onto agar and incubated for 24 hr at 37^o^C.

**Statistical analysis**

Data are expressed as mean±SD of three experiments. Statistical analyses were performed using SPSS version 16. Statistical significance was determined using Student’s t-test for comparisons between treated versus control cells and a p<0.05 was considered statistically significant.

## Results

**Cytotoxicity evaluation of methanol extracts on human cancer and normal cells**

**Figure 1:**
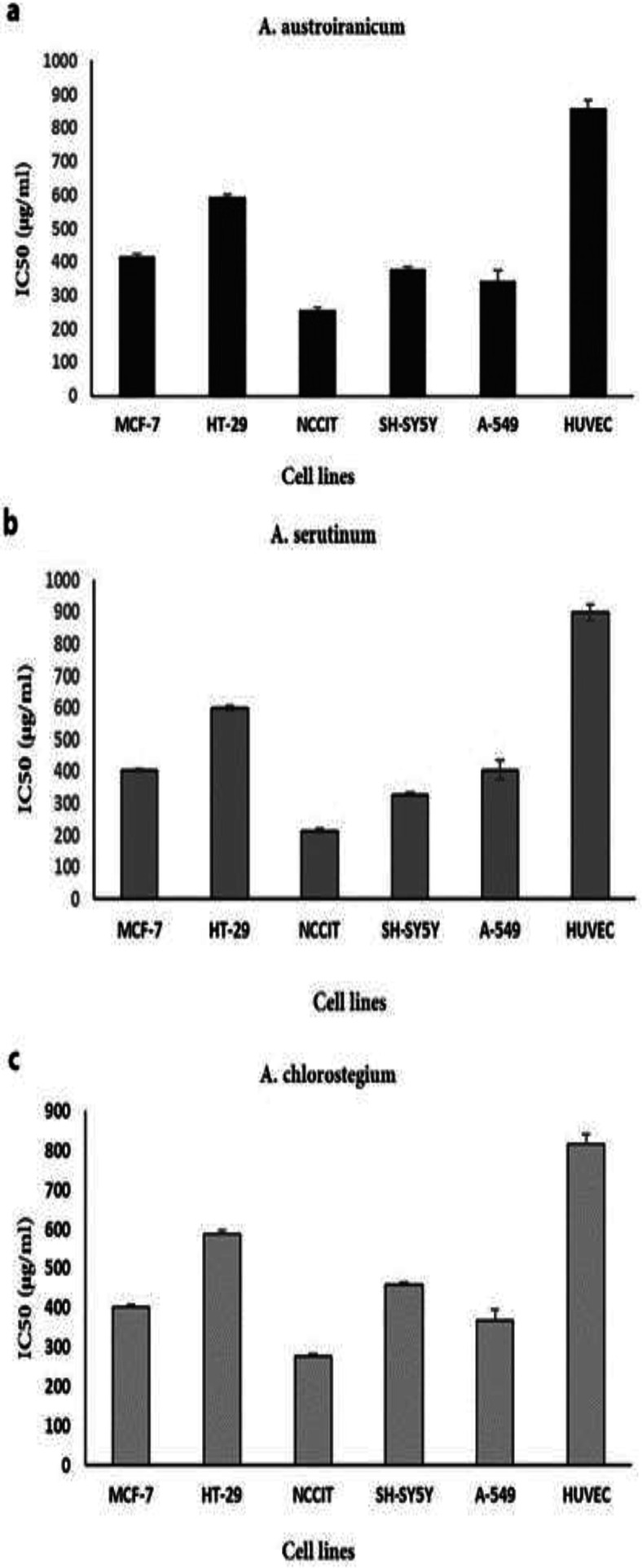
Anti-proliferative effects of methanol extract of *A. austro-iranicum* (a), *A. serotinum* (b) and *A. chlorostegium* (c) on various tumor cells (MCF-7, HT29, NCCIT, SH-SY5Y and A549) and one normal (HUVEC) cell line. Cells were treated with various concentrations of the extracts (10, 20, 40, 80, 160, 320, 640, and 1280 µg/ml) for 48 hr and subjected to MTT assay. IC_50_ values are expressed as mean±SD of three independent experiments

The IC_50_ values of all three extracts were determined against both tumor and non-tumor cells (Figure 1). 

NCCIT was the most susceptible cell to treatment with all three extracts with an IC_50_ value of 213 µg/ml for *A. serotinum*, 257 µg/ml for *A. austro-iranicum* and 277 µg/ml for *A. chlorostegium*.  The lowest cytotoxic effect was found against normal cell line compared to cancer cell lines with IC_50_ values of 858 µg/ml for *A*.* austro-iranicum*, 817 µg/ml for *A. chlorostegium* and 900 µg/ml for *A. serotinum. *The MTT assay showed that methanol extracts are more toxic to human cancer cell lines (MCF-7, A549, HT29, SH-SY5Y and NCCIT) compared to the normal cell line HUVEC. Since *A. serotinum* extract showed higher cytotoxic activity against NCCIT cells, it was used for further investigation of *in vitro* anticancer activity, apoptosis induction and cell cycle arrest.

**Methanol extract of** ***A. serotinum*** **suppressed colony formation of NCCIT cells**

**Figure 2:**
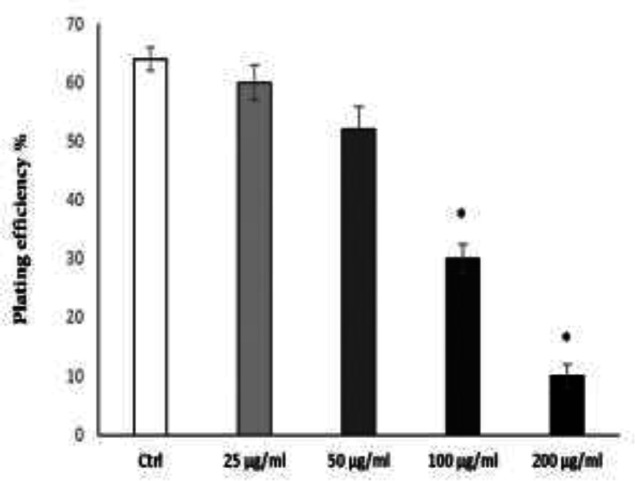
Methanol extract of *A. serotinum* decreased the colony formation of NCCIT cells. NCCIT cells were seeded into 6-well plates at a density of 1000 cells/well. After 24 hr, the cells were treated with the indicated concentrations of extract for 14 days. Cells were fixed and stained with crystal violet to visualize the colonies for counting. Colony numbers (≥50 cells per colony) were counted. The experiments were independently repeated 3 times and plating efficiency ±SEM was calculated. *p<0.05 shows significant differences as compared to the control (ctrl) as tested by the student’s t-test

Capacity of a single cell to proliferate into a colony can be tested by clonogenic assays. We tested clonogenicity of NCCIT cells as embryonal carcinoma cells, after treatment with methanol extract of *A. serotinum*. Methanol extract dose-dependently inhibited colony formation ability of NCCIT cells. The minimum concentration of the extract (25 µg/ml) caused a reduction of approximately 4% in terms of colony formation and higher concentrations (100 and 200 µg/ml) significantly abrogated colony formation of NCCIT cells (Figure 2). Therefore, the inhibitory effect of *A. serotinum* extract on colony formation of NCCIT, confirmed its cytotoxic effect evaluated by MTT test.

**Methanol extract of** ***A. serotinum*** **promoted cell death by apoptosis**

**Figure 3:**
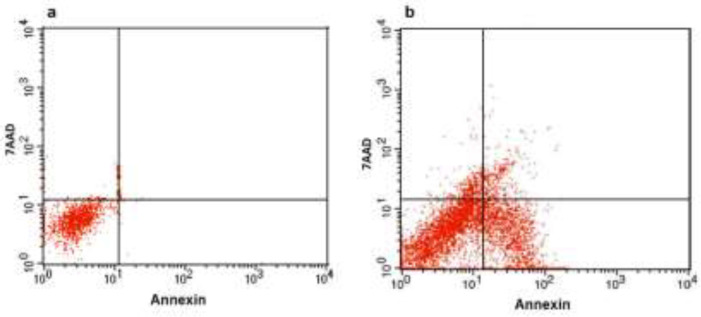
Methanol extract of *A. serotinum* stimulated apoptosis in NCCIT cells. Apoptosis cells were evaluated using flow cytometry following annexin V-PE and 7-AAD staining in non-treated control cells (a) and plant extract-treated cells (b). Cells in the lower left quadrant are viable, those in the lower right quadrant are early apoptotic and those in the upper right and left quadrant are late apoptotic and necrotic

Apoptosis induction in NCCIT cells after 48 hr treatment with methanol extract of *A. serotinum *was monitored by ﬂow cytometry using annexin V-PE/7-AAD kit. Marker for early apoptosis is translocation of phosphatidylserine to the exterior surfaces of the plasma membrane which can be detected by annexin V-PE binding. Late apoptotic or necrotic cells are permeable for 7-AAD, and DNA stains with this reagent. After treatment with 700 μg/mL *A. serotinum* extract for 48 hr, the early apoptotic rate was 36% and late apoptotic rate was 5% (Figure 3). 

**Methanol extract of** ***A. serotinum*** **induced G1 cell cycle arrest in NCCIT cells**

**Figure 4:**
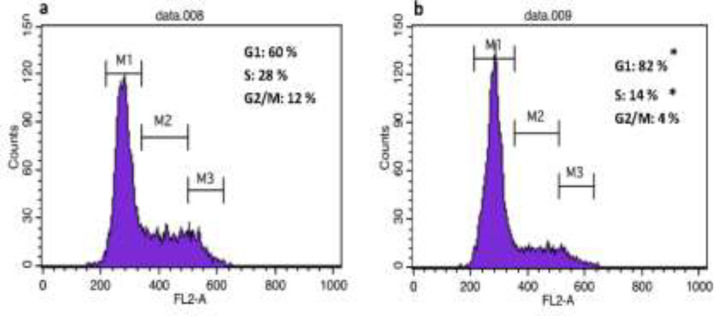
The effect of *A. serotinum* methanol extract on cell cycle progression in NCCIT cells. Untreated cells (a) and cell incubated for 48 hr in the presence of *A. serotinum* extract (b) were harvested by trypsinization, fixed, and stained with propidium iodide to determine the profile of the cell cycle by flow cytometry. Results are expressed as mean±SD of 3 independent experiments. *p<0.05 shows significant differences as compared to the control as tested by the Student’s t-test. Each DNA histogram represents one of the three independent experiments

To investigate whether methanol extract could induce cell cycle perturbations in cancer cells, propidium iodide-stained nuclei were analyzed using flow cytometry. As shown in Figure 4, 48 hr treatment with extract of *A. serotinum* (300 µg/ml) increased the proportion of NCCIT cells in G0/G1 phase (20%) and reduced the proportion of S-phase cells (14%) compared to the control, indicating that the extract induces G0/G1 cell cycle arrest in NCCIT cells.

**Antioxidant activity**

The scavenging activity of the antioxidants can decrease oxidative stress and control some diseases including cancer, AIDS and neurodegenerative conditions. To assess antioxidant potential of the examined plant extracts, both DPPH scavenging assay and measurement of intracellular ROS as commonly used assays for antioxidant studies of specific compounds or extracts, were used (Amarowicz et al., 2004 ▶). 

**DPPH Free Radical Scavenging Activity**

DPPH assay is the most commonly method to determine the antioxidant activity of different components (Kumar et al., 2008 ▶). As shown in Table 1, DPPH test confirmed that methanol extracts of *A. austro-iranicum*, A*. serotinum *and *A. chlorostegium* exerted approximately the same free radical-scavenging activity with IC_50 _values of 30-37 µg/ml. 

Extracts that possess IC_50_ values ranging between 10 and 50 mg/ml are considered to have strong antioxidant activities (Phongpaichit et al., 2007 ▶; Jadid et al., 2017 ▶). Therefore, methanol extracts of three species of *Acantholimon* that were tested in this study, possess strong antioxidant activities. 

**Intracellular reactive oxygen species scavenging activity**

**Figure 5:**
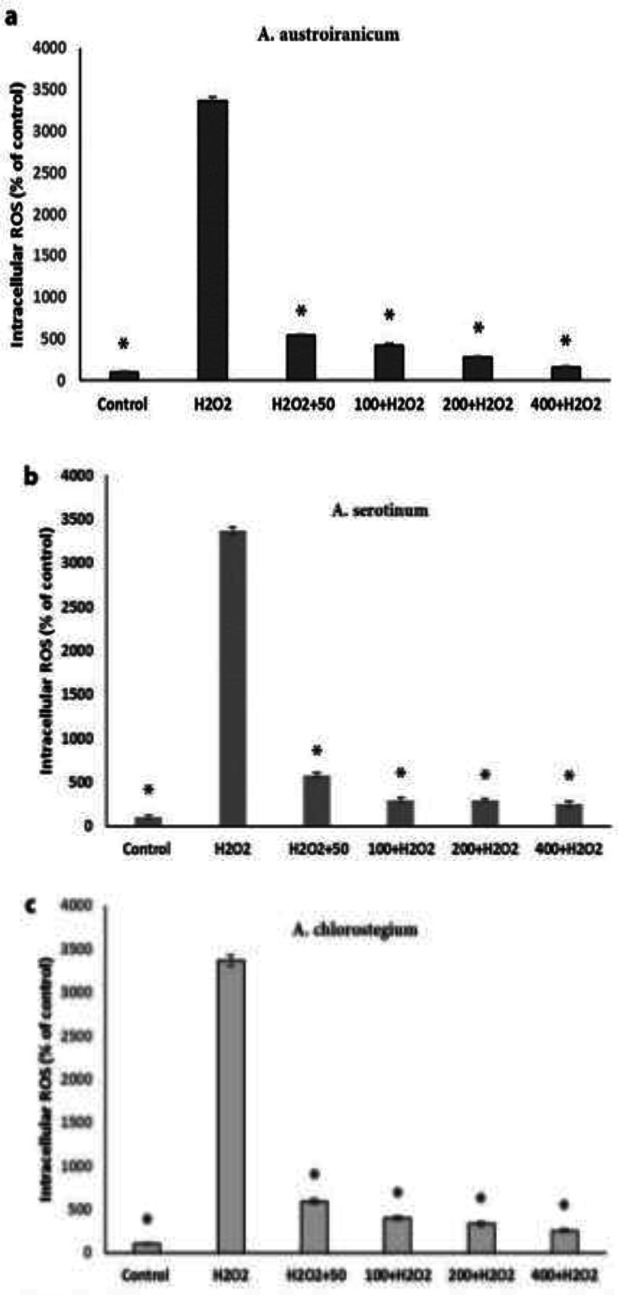
Effect of the methanol extracts from *A. austro-iranicum, A. serotinum *and* A. chlorostegium* on ROS production in NCCIT cells exposed to H_2_O_2_. Cells were treated with H_2_O_2_ (200 μM) in the presence or absence of each plant extract at different concentrations (50, 100, 200 and 400 µg/ml). Data are means±SEM of three independent experiments. *p<0.05 as compared to H_2_O_2_-treated cells

To investigate antioxidant potential of methanol extracts of three species of *Acantholimon*, the intracellular ROS levels were measured in control, H_2_O_2_-treated and H_2_O_2_-treated cells incubated with different concentrations of the plant extracts. As shown in Figure 5, exposure of NCCIT cells to H_2_O_2_ (200 µM) led to an increase in the fluorescent intensity (33-fold) as compared to the control cells which showed production of ROS by H_2_O_2_. 

**Table 1:** The inhibitory concentration 50% (IC_50_) of methanol extract of *A. austroiranicum*, *A. serotinum* and *A. chlorostegium* in DPPH test (Mean±SD).

	***A. austroiranicum*** ** (µg/ml)**	***A. serotinum*** ** (µg/ml)**	***A. chlorostegium*** ** (µg/ml)**	**BHT (µg/ml)**
**IC**_50_	37±3.5	30±1.5	34±1.08	7.45±1.08

**Table 2:** Antibacterial activity of the methanol extract of *A. austro-iranicum, A. festucaceum *and *A. chlorostegium*

	***A. austroiranicum ***			***A. serotinum ***			***A. chlorostegium***			**ciproﬂoxacin**		
**Test Bacteria**	**D.D**	**MIC**	**MBC**	**D.D**	**MIC**	**MBC**	**D.D**	**MIC**	**MBC**	**D.D**	**MIC**	**MBC**
***E. faecalis ***	7	50	100	7	50	100	6	25	50	26	8	16
***S. aureus ***	3	25	50	4	25	50	4	25	50	28	8	16
***P. aeruginosa ***	13	6.25	12.5	11	12.5	25	11	3.175	6.25	32	8	16
***E. coli ***	11	0.39	0.78	13	0.78	1.56	12	3.175	6.25	30	0.25	0.5

D.D: Inhibition zone in diameter (mm) around the impregnated discs. MIC: Minimal inhibition concentrations (as mg/ml). MBC: minimal bactericidal concentration (as mg/ml)

However, co-treating with H_2_O_2_ (200 µM) and different concentrations (50, 100, 200 and 400 µM) of each plant extract, reduced fluorescent intensity around to 13 folds as compared to H_2_O_2-_treated cells that indicated that methanol extracts act as ROS scavenger and have significant antioxidant activities. 

**Antibacterial activity**

The antibacterial activity of *A. austro-iranicum*, *A. serotinum* and *A. chlorostegium* methanol extract was examined both qualitatively and quantitatively against two Gram-positive and two Gram-negative bacteria. The results presented in Table 2 were compared with ciproﬂoxacin. Inhibition zones and MIC values for bacterial strains were in the range of 3-13 mm and 0.39-50 mg/ml, respectively. The extracts showed higher inhibitory effects against *E. coli* and *P. aeruginosa* as Gram-negative bacteria with inhibition zones of 11-13 mm and MIC range of 0.39-12.5 mg/ml and weaker antibacterial activity against Gram-positive bacteria *E. faecalis* and *S. aureus* with inhibition zones of 3-7 mm and MIC range of 25-50 mg/ml. Variation in the cell wall structure between Gram-negative and Gram-positive may have led to different inhibitory effects. 

## Discussion

Pharmacological properties that were described for the Plumbaginaceae family plants include anticancer, anti-inflammatory, antioxidant, anti-mycobacterial, antimicrobial, antiatherogenic, cardiotonic, neuroprotective and insecticidal activities (Oyedapo, 1996 ▶; Kuo et al., 2002 ▶; Chaung et al., 2003 ▶; Mossa et al., 2004 ▶; Nguyen et al., 2004 ▶; SivaKumar et al., 2005 ▶; Yang et al., 2010 ▶; Dhale and Markandeya, 2011 ▶; Eren, 2016 ▶; Lashgari et al., 2016 ▶; Sundari et al., 2017 ▶).

For example, study on anti- proliferative effects of some species of the Plumbaginaceae (e.g. *Acantholimon longiscapum, Plumbago rosea *and *Plumbago zeylanica*) indicated good and moderate levels of tumor inhibition and cancer cell cytotoxicity (Ahmad et al., 2008 ▶; Nabi et al., 2013 ▶; Anuf et al., 2014 ▶; Sharma and Kaushik, 2014 ▶; Roy et al., 2017 ▶; Sundari et al., 2017 ▶). Antioxidant potential was also detected in some species of the genus *Plumbago* (Plumbaginaceae) such as *P. zeylanica* and *P. rosea* which have a high level of phenolic content (Nahak and Sahu, 2011 ▶; Sundari et al., 2017 ▶). Antimicrobial activity *P. zeylanica, P. scandens* and *P. indica *which belong to the family Plumbaginaceae, toward drug resistant and pathogenic bacteria were also indicated in some reports (Paiva et al., 2003 ▶; Rahman and Anwar, 2007 ▶; Dhale and Markandeya, 2011 ▶; Saha and Paul, 2014 ▶; Sharma and Kaushik, 2014 ▶; Shukla et al., 2016 ▶). Different parts of these plants are used to treat various diseases (Thakur et al., 1989 ▶; Tilak et al., 2004 ▶). Moreover, the hepatoprotective effect and their usage in the treatment of liver disease have also been demonstrated in some researches (Girish and Pradhan, 2012 ▶; Rahmatullah et al., 2012 ▶; Nasiri et al., 2016 ▶; Gazor et al., 2017 ▶). According to the literature, plants from the Plumbaginaceae contain various secondary metabolites and active constituents such as alkaloids, glycoside, reducing sugars, simple phenolic, tannins, lignin, saponins, anthocyanin, quinines and flavonoids that contribute to their biological activities (Asen and Plimmer, 1972 ▶; Gunaherath et al., 1983 ▶; Duffey and Stout, 1996 ▶; Sreelatha et al., 2010 ▶; Dhale and Markandeya, 2011 ▶; Trabelsi et al., 2012 ▶; Pavela, 2013 ▶). For example, it was observed that one of the major secondary metabolites present in members of the Plumbaginaceae is plumbagin (Babula et al., 2005 ▶; Jeyachandran et al., 2009 ▶; Sharma and Kaushik, 2014 ▶). Several pharmacological activities, e. g. antitumor, antimicrobial, insecticidal, would healing, anti- inflammatory and antifertility actions, have been identified for plumbagin (Kavimani et al., 1996 ▶; Kini et al., 1997 ▶; Reddy et al., 2002 ▶; Sheeja et al., 2010 ▶). The genus *Acantholimon *may also contain plumbagin which needs to be determined by phytochemical screening. 

In conclusion, our study can be considered the first study on anti-cancer, antioxidant and antibacterial potential of three Iranian endemic species of *Acantholimon* including *A. austro-iranicum, A. serotinum* and *A. chlorostegium. *The results showed a specific cytotoxic effect for the extracts against cancerous cells. Moreover, the significant antioxidant potential shown by the extracts demonstrates their ability to protect the non-target (normal) cells against oxidative stress and antibacterial tests indicated good activity of extracts against Gram-negative bacteria. Unexplored genus *Acantholimon* needs more attention for detailed scientific investigations. For instance, there is no evidence in the literature about chemical constituents of *Acantholimon. *Therefore, phytochemical studies for identification and elucidation of active constituents of the extracts from the three examined species of *Acantholimon* are needed. 
